# Evaluation of the in vitro effects of commercial herbal preparations significant in African traditional medicine on platelets

**DOI:** 10.1186/s12906-019-2644-z

**Published:** 2019-08-22

**Authors:** Mmamosheledi E. Mothibe, Christina P. Kahler-Venter, Elzbieta Osuch

**Affiliations:** 1 Division of Pharmaceutical Sciences, School of Pharmacy, Sefako Makgatho Health Sciences University, Molotlegi Street, Ga-Rankuwa, Pretoria, South Africa; 2 Department of Pharmacology and Therapeutics, School of Medicine, Sefako Makgatho Health Sciences University, Molotlegi Street, Ga-Rankuwa, Pretoria, South Africa

**Keywords:** African traditional medicine; herbal medicine; human platelets, Luminescence, PMA, fMLP

## Abstract

**Background:**

Commercial herbal medicines (CHMs) marketed as immune boosters are gaining wide popularity in South Africa, in the absence of control and regulatory guidelines. These commercially packaged and labelled herbal preparations, acquired in various retail outlets, are used without consulting either a conventional health provider or a traditional health practitioner. Although they are indicated for immune-boosting purposes, they might exert many other beneficial and unwanted effects on physiological systems. Platelets are crucial in haemostasis and important for the immunological system. The aim was to investigate the effect of the CHMs used to strengthen the immune system on the activity of human platelets.

**Methods:**

Six CHMs commonly used as African traditional medicines in Pretoria, South Africa, were tested for their effects on healthy, isolated human platelets, using a bioluminescence method. The tested herbal medicines were *Intlamba Zifo™*, *Maphilisa*™ Herbal medicine, *Matla*™ African medicine for all diseases, *Ngoma*™ Herbal Tonic Immune Booster, *Stametta*™ Body Healing Liquid, and *Vuka Uphile*™ Immune Booster and serial-diluted standards of each from 10 to 10,000 times. The luminol-enhanced luminescence activity of the platelets was measured after incubation with the herbal medicines and activation with phorbol myristate acetate (PMA) or N-formyl-methionyl-leucyl-phenylalanine (fMLP).

**Results:**

Five herbal medicines, namely *Intlamba Zifo™*, *Maphilisa*™ Herbal medicine, *Matla*™ African medicine for all diseases, *Stametta*™ Body Healing Liquid, and *Vuka Uphile*™ Immune Booster exerted comparable weak inhibitory effects on both PMA and fMLP-induced platelets, which were concentration dependent at high doses, and inversely related to concentration at low doses. *Intlamba Zifo™*, *Matla*™ African medicine for all diseases, *Stametta*™ Body Healing Liquid, and *Vuka Uphile*™ exhibited weak, but non-systematic stimulatory effects at low doses, which were not statistically significant. *Ngoma*™ Herbal Tonic Immune Booster had weak, inhibitory effects at high doses and weak stimulatory effects that were inversely related to concentration at low doses.

**Conclusion:**

The findings suggest a potential beneficial role of the CHMs in the suppression of platelets’ reactivity and in enhancing the immune system. Caution, however, should be exercised as platelet inhibition and stimulation predispose to the risk of bleeding and thrombosis, respectively.

## Background

According to reports, a significant section of the population in South Africa (SA) uses the dual healthcare system, in which both the traditional and orthodox medicines are sought, depending on the sickness [1]. Herbal medicines (HMs), commonly used as traditional medicine (TM), are plant-based medicines either obtained from a traditional health practitioner or self-prepared as home remedies [2]. Currently, in SA, there is a growing use of commercially prepared herbal medicines sold in many retail outlets. These commercially packaged and labelled herbal preparations used as African TM (ATMs) are not regulated. There has been an upsurge of commercial herbal medicines (CHMs) that claim to strengthen the immune system. The CHMs are widely popular and are commonly found in various retail outlets such as *muthi* shops, pharmacies, health shops and some grocery stores. The CHMs in this study were part of the 10 most commonly sold in retail pharmacies in Pretoria, Gauteng, SA; and were selected on their use and commercial importance. They were indicated as immune boosters or intended to strengthen the immune system or the body [3]. It was perceived that the market for CHMs could be driven by the desire for urbanised populations to use TM but not having adequate time and means to produce the medicines. The self-medication with CHMs could also be from the perception that, as natural products, they signify purity, simplicity, and safety, and for the convenience of their availability without a prescription [4]. This market, therefore, will keep growing, as it is convenient regarding cost and time. Currently, in SA, there are no guidelines that govern the provision of information of the CHMs used as ATMs either on the label or package insert of these herbal mixtures. The information presented is solely at the discretion of the manufacturer and there, therefore, is no consistency in presentation.

CHMs can be described as products with medicinal properties that contain two or more plants or herbs that can act alone, in synergy, or additively in the restoration or maintenance of health [5]. In their use for immune-boosting purposes, the CHMs could affect many physiological systems and, therefore, inherently exert many other effects. One of the critical elements in physiology is platelets, and the functioning of these platelets could be affected by the many chemical molecules in CHMs through different biochemical pathways. Platelets are crucial in haemostasis and are significantly involved in the inflammatory and the immune responses [6, 7]. Studies have shown that they participate in immunoregulation by secreting chemical mediators and interacting with various immune and endothelial cells. They are furthermore regarded as potent effector cells of the innate immune system, while also acting as mediators between the innate and adaptive immune responses [8]. They are regarded as essential markers in disease pathophysiology, including cardiovascular [9] and inflammatory disorders [6, 8].

Platelet activity is modulated by various cell-surface receptors, as well as chemicals that directly stimulate intracellular biochemical molecules. Activated platelets release chemical substances such as reactive oxygen species (ROS), which include the superoxide anion (O_2_^−^), hydrogen peroxide (H_2_O_2_), and hydroxyl radicals (OH^−^) [9, 10]. Platelet ROS play a direct role in thrombosis [9] and directly and indirectly enhance the immune and inflammatory system [6, 8, 11]. Production of ROS because of platelet NADPH oxidase (NOX) action results in the emission of a burst of light termed chemiluminescence (CL), which can be measured in a luminometer [10]. The effects of common Western herbal medicines on platelets are known, and some of their mechanisms are well understood. This information is lacking for medicinal plants and herbal mixtures used as African traditional medicines.

The CHMs in this study contain some well-known African medicinal plants, including *Aloe ferox, Siphonochilus aethiopicus, Hypoxis hemerocallidea, Sutherlandia frutescens*, and *Harpagophytum procumbens*. The ethnobotanical uses of these plants are widespread, ranging from minor illnesses such as colds and headaches to serious conditions such as gastric ulcers and cancer. Various pharmacological and biological activities of the plants have been reported, including anti-inflammatory, immunomodulatory, and antioxidant activities [12]. There is no information about their activity on platelets. We investigated the effects of commonly used commercially available herbal mixtures on platelets using chemiluminescence measurements. We also explored whether the effects were mediated via a receptor-based or a non-receptor-based mechanism.

## Methods

### Reagents and instruments

A chemiluminescence assay, including the reagents used, was performed as described by Mothibe et al. [13] in an Orion™ L Microplate Luminometer supplied by Berthold Detection Systems.

### The commercial herbal medicines and preparation

**Fig. 1:**
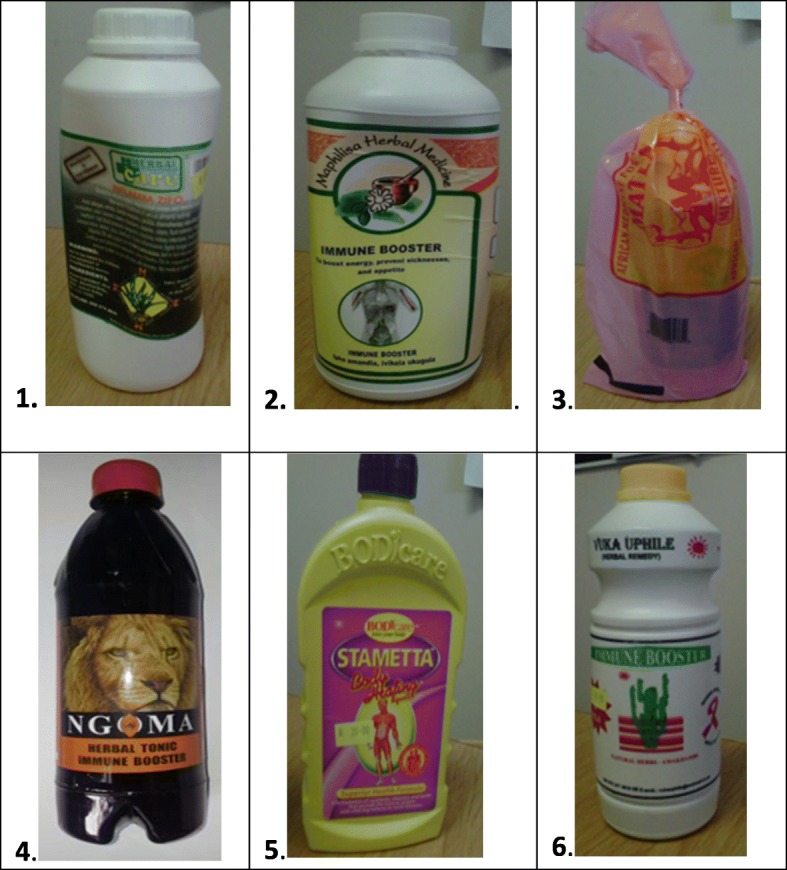
The selected commercial herbal medicines. 1. Intlamba zifo™ (HM1), 2. *Maphilisa™* Herbal Medicine (HM2), 3. *Matla™* African medicine for all diseases (HM3), 4. *Ngoma*™ Herbal Tonic Immune Booster (HM4), 5. *Stametta*™ Body healing liquid (HM5), *Vuka Uphile™* Herbal remedy (HM6)

The CHMs selected were bought over the counter from a local pharmacy in Pretoria. The six CHMs denoted HM1 to HM6 were as follows: *Intlamba Zifo™* (HM1), *Maphilisa*™ Herbal medicine (HM2), *Matla*™ African medicine for all diseases (HM3), *Ngoma*™ Herbal Tonic Immune Booster (HM4), *Stametta*™ Body Healing Liquid (HM5), and *Vuka Uphile*™ Immune Booster (HM6). Four serial dilutions of each of the six herbal mixtures were prepared, from 10x, 100x, 1000x to 10,000x with Hanks’ balanced salt solution (HBSS). Each neat herbal mixture (the undiluted CHM) and its diluted solutions were tested in triplicate in the luminometer. Figure 1 displays the pictures of the CHMs, and Table 1 shows the composition of each HM as it appears on the packaging or the product label.

**Table 1:** The components of each herbal medicine as listed on the package or label

Herbal mixture	Ingredients
*Intlamba zifo™* (HM1)	Active: aloe, water, assorted herbs, H_2_O_2_, sunset yellow No 6-CI 15985
*Maphilisa™* Herbal Medicine (HM2)	African potato, *Aloe ferox*, African ginger, citric acid, Sutherlandia and wild olive. Preserved with sodium benzoate
*Matla™* African medicine for all diseases (HM3)	None listed
*Ngoma™* Herbal Tonic Immune Booster (HM4)	Sutherlandia (cancer bush), Echinacea, dandelion, alfalfa-lusern, aloe ferox, harpagophytom (devil’s claw), alcohol (13,5%)
Stametta™ Body healing liquid (HM5)	Aloe 1.667 g, ascorbic acid 1.667 g, aniseed oil 0.1817 g, magnesium sulphate 71.667 g in each 500 ml Preservative: nipastat 0.02% m/v.
*Vuka Uphile™* Herbal remedy (HM6)	Natural herbs

### Collection of blood samples

A qualified professional nurse collected blood samples from healthy adult volunteers. The self-declared healthy volunteers included males and females aged between 20 and 31 years. They were non-smokers, not on any prescribed medication, and had not been on self-medication, supplements, or any herbs or herbal mixtures for the 2 weeks before blood sample donation. Written information about the study was provided. Each volunteer signed a consent form after being informed of voluntary participation and guaranteed confidentiality of their details and the results. All ethical requirements were adhered to, including anonymity during the handling of samples.

#### Isolation of platelets

Approximately 10 ml of blood was collected from each volunteer in tubes with citrate preservative. Each sample was centrifuged at 1000 rpm (250 g) for 10 min. The plasma rich in platelets (PRP) was collected, onto which 40 μl of citric acid was added, mixed well and centrifuged at 2200 rpm (1000 g) for 10 min. After discarding the supernatant, the pellet was suspended in 4 ml of a tyrode plus ethylene diamine-tetra-acetic acid (EDTA) solution and allowed to stand for 10 min at room temperature. The suspension was centrifuged at 2200 rpm (1000 g) for six minutes. The supernatant was discarded, and the tyrode solution was added to the pellet to a final volume of 6 ml. A platelet count was performed at the Haematology division of the National Health Laboratory Services.

### Luminescence procedure

The experimental process was as outlined by Mothibe et al. [13]. Briefly, each test well of a 96-well microplate was filled with 25 μl of the test solution of each HM and 25 μl of the platelet suspension. The control wells included 25 μl Hanks Balanced Salt Solution (HBSS) and 25 μl of the platelet suspension. Luminol (25 μl) was added to all the wells for amplification of the light signal. A 25 μl volume of either N-formyl-methionyl-leucyl-phenylalanine (fMLP) or phorbol myristate acetate (PMA) and HBSS was added to a final volume of 200 μl in each well. Luminol-enhanced luminescence (LEL) activity measurements of each sample were taken over a period of 60 min.

#### Data collection and handling

The platelet counts were corrected to 1 × 10^3^ cells/μl for the LEL activity measured. The average LEL activity of the platelets was calculated per time interval, and the percentage inhibitions (% inh) for each HM standard was calculated and compared to the controls using the formula: $\%\mathrm{inh}=\frac{\left({\mathrm{RLU}}_{\mathrm{ctrl}}\hbox{-} {\mathrm{RLU}}_{\mathrm{test}}\right)}{{\mathrm{RLU}}_{\mathrm{ctrl}}}\times 100$

where RLU_ctrl_ is the relative luminescence units (RLU) of the control, and RLU_test_ is that of the test samples. The % inhibitions were graded as weak (< 50%), moderate (50–79%) and potent (≥ 80%). This grading was adopted from a similar chemiluminescence study by Koko et al. [14]. For statistical analysis, the Microsoft Excel™ data analysis tool, t-Test Two sample Assuming Unequal Variance, was used. Where the *p*-value was less than or equal to 0.05, the results were declared as statistically significant.

## Results

**Fig. 2:**
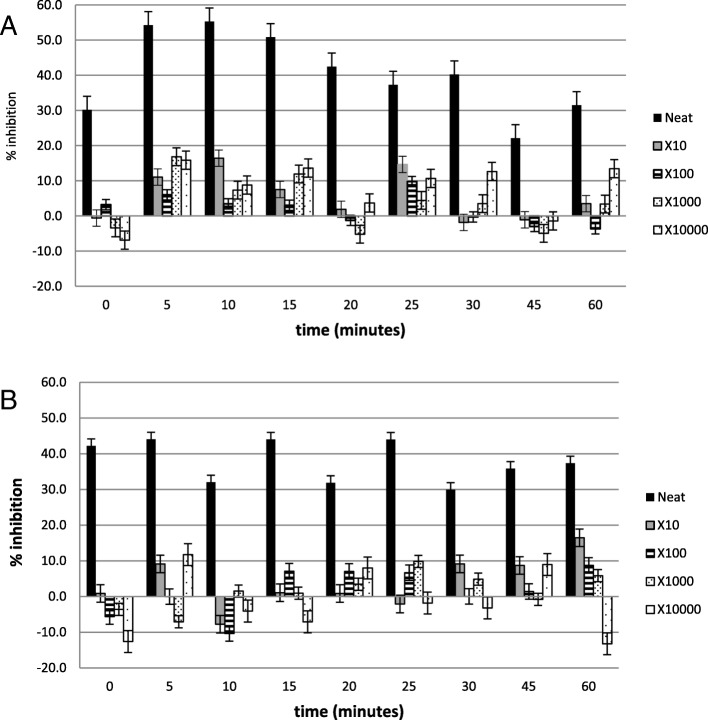
The % inhibition of platelets after incubation with the neat HM1 and diluted standards, and activation with (**a**) fMLP and (**b**) PMA

**Fig. 3:**
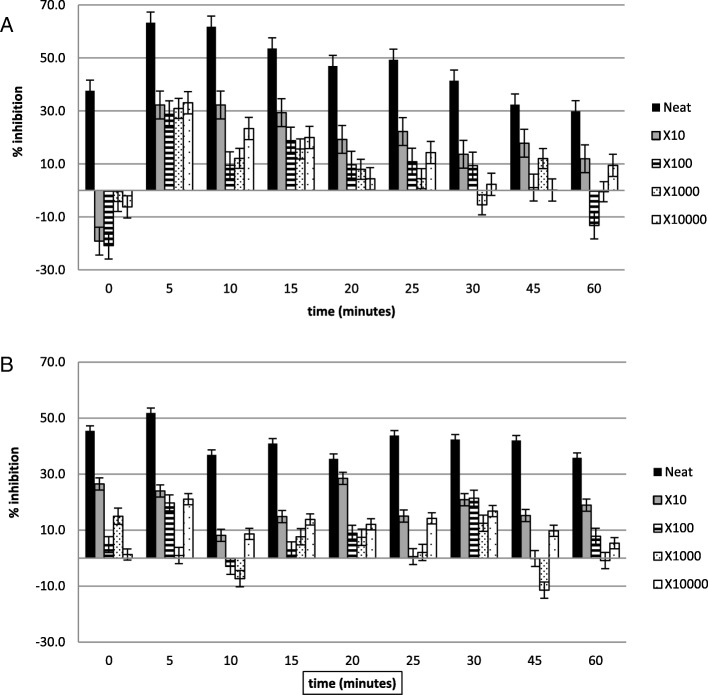
The % inhibition of healthy platelets after incubation with the HM2 and diluted standards, and activation with (**a**) fMLP and (**b**) PMA

**Fig. 4:**
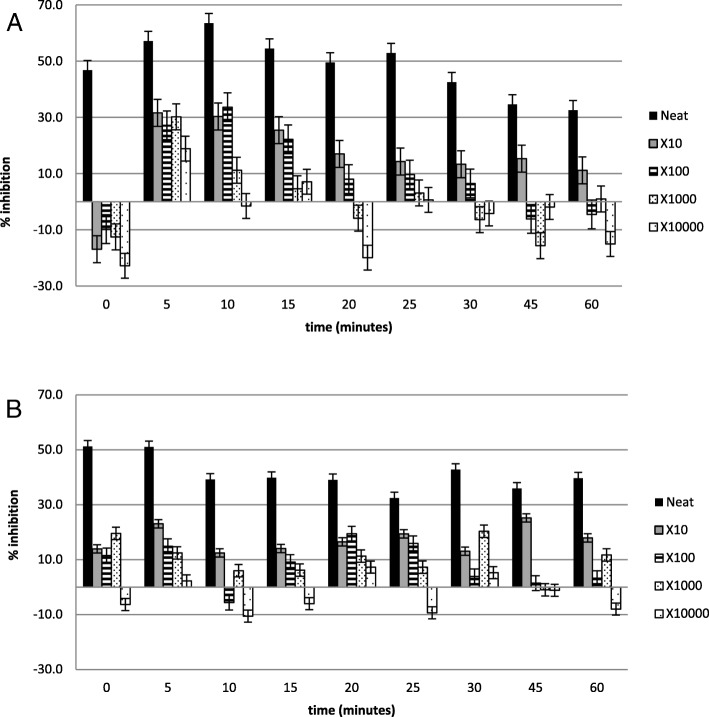
The % inhibition of healthy platelets after incubation with the HM3 and diluted standards, and activation with (**a**) fMLP and (**b**) PMA

**Fig. 5:**
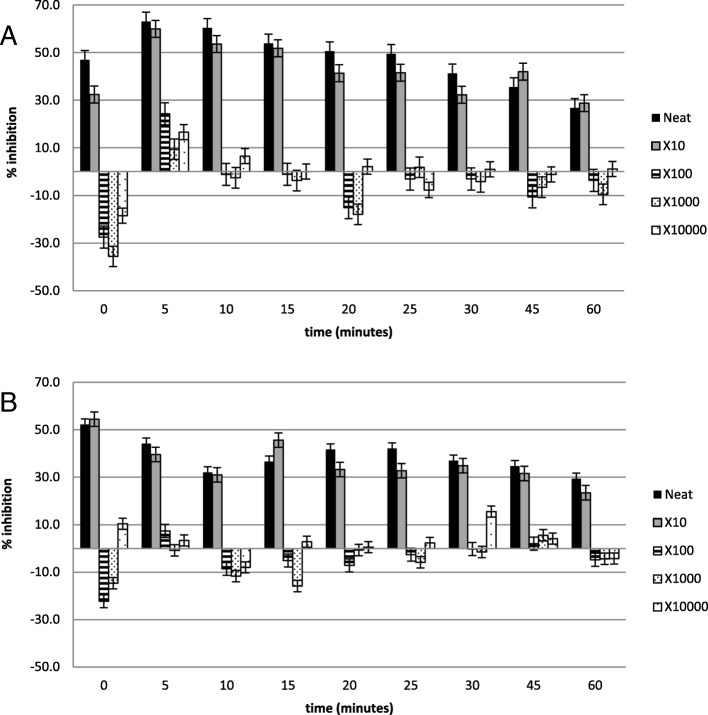
The % inhibition of healthy platelets after incubation with the HM4 and diluted standards, and activation with (**a**) fMLP and (**b**) PMA

**Fig. 6:**
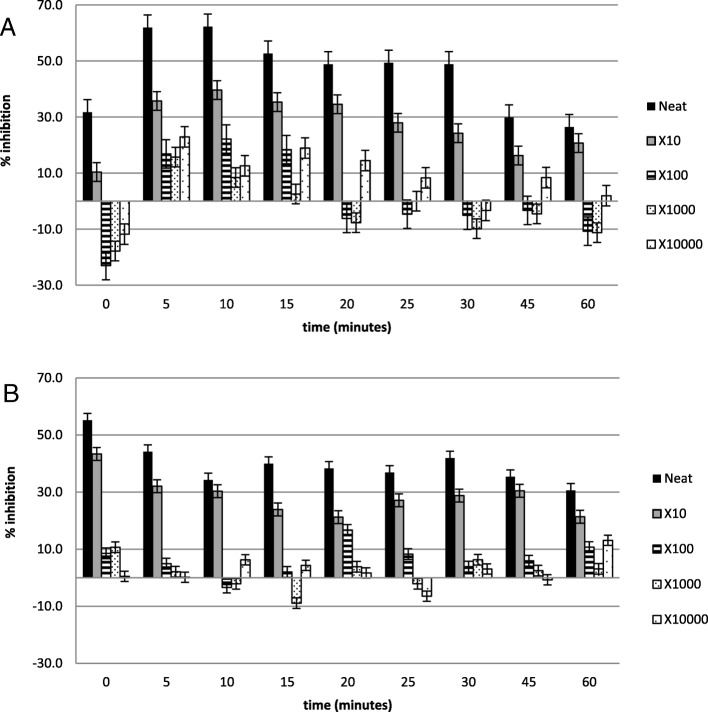
The % inhibition of healthy platelets after incubation with the HM5 and diluted standards, and activation with (**a**) fMLP and (**b**) PMA

**Fig. 7:**
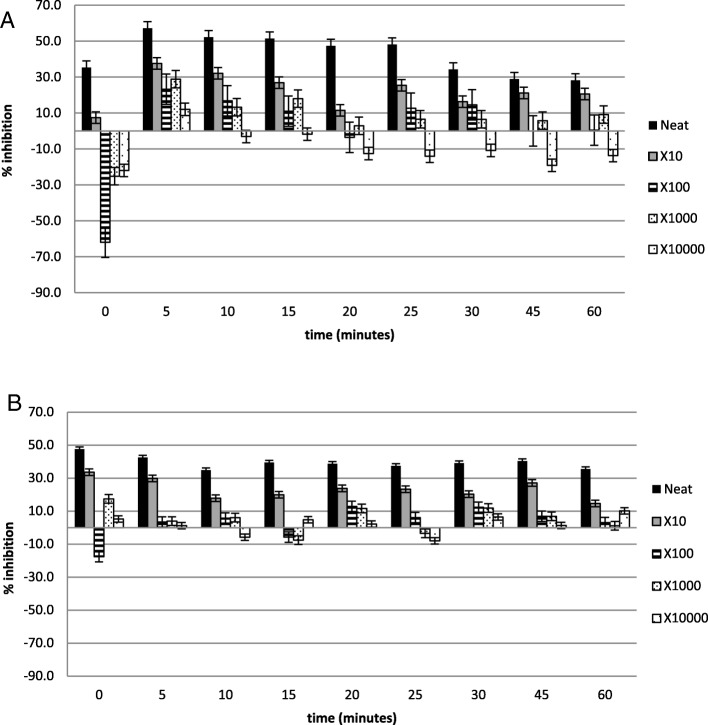
The % inhibition of healthy platelets after incubation with the HM6 and diluted standards, and activation with (**a**) fMLP and (**b**) PMA

Figures 2, 3, 4, 5, 6 and 7 show the percentage inhibitions of the platelets at various time intervals exposed to each HM and its diluted standards, after they were activated with either fMLP (A) or PMA (B). Positive values (above zero) indicate inhibition effect and negative ones indicate stimulation effect. Each point is displayed as mean ± SD, and *n* = 8.

The HMs exerted inhibitory effects on both PMA- and fMLP-induced platelets, sustained over 60 min. When analysed per time interval, the effect ranged from weak to moderate inhibition (Figs. 2, 3, 4, 5, 6 and 7), with the magnitudes varying with the different concentrations of the HMs. There was also weak stimulation for all HMs at lower concentrations, except HM6, which was also moderate at the lower concentrations (Fig. 7).

Table 2 shows the calculated average % inhibitions of the HMs. The weak inhibitory effects were generally concentration-dependent when analysed from the neat, the 10x and 100x diluted standards of HM1, HM2, HM3, HM5, and HM6. When observed in the lower concentration standards (100x, 1000x and 10,000x diluted standards), the inhibitory effects were inversely related to the concentration for HM1 and HM2 in fMLP-induced platelets (A), and for HM1 (A), HM2 and HM5 (B) when studied between the 1000x and the 10,000x diluted standards. The stimulatory effects, when present, were non-systematic and were statistically not significant. The HM4 showed inhibitory effects in the neat and 10x diluted standards, and weak stimulatory effects, inversely related to the concentration in the 100x and 1000x standards.

**Table 2:** The average % inhibitions in 60 min of platelets incubated with the HMs

Extracts tested	Strength of extract	% inhibition	
		A: fMLP-induced platelets	B: PMA-induced platelets
HM1- *Intlamba Zifo™*	Neat	40.5 ± 11.5*	38.0 ± 5.8*
	10x dilution	5.7 ± 7.0	4.1 ± 7.4
	100x dilution	1.9 ± 4.4	1.7 ± 6.5
	1000x dilution	3.7 ± 7.6	- 1.7 ± 5.1
	10,000x dilution	7.8 ± 7.7	1.5 ± 9.2
HM2- *Maphilisa*™ Herbal medicine	Neat	46.2 ± 12.0*	41.6 ± 5.2*
	10x dilution	17.7 ± 15.8*	19.1 ± 6.5*
	100x	6.0 ± 15.2	7.0 ± 8.6*
	1000x	8.1 ± 11.4	2.9 ± 8.7
	10,000x	11.2 ± 12.6	11.4 ± 6.0
HM3- *Matla*™ African medicine for all diseases	Neat	48.2 ± 10.3*	41.3 ± 6.3*
	10x dilution	15.7 ± 14.4*	17.3 ± 4.5*
	100x dilution	9.6 ± 15.3	8.2 ± 8.1
	1000x dilution	1.1 ± 13.9	10.4 ± 6.8
	10,000x dilution	−4.3 ± 13.3	− 2.9 ± 6.6
HM4- *Ngoma*™ Herbal Tonic Immune Booster (HM4),	Neat	47.5 ± 11.6*	38.9 ± 7.0*
	10x dilution	42.6 ± 10.7*	36.3 ± 9.1*
	100x dilution	−4.6 ± 13.8	− 4.5 ± 8.2
	1000x dilution	−7.7 ± 12.8	−5.5 ± 7.2
	10,000x dilution	0.0 ± 9.5	3.0 ± 7.0
HM5- *Stametta*™ Body Healing Liquid	Neat	45.8 ± 13.4*	39.7 ± 7.1*
	10x dilution	27.2 ± 10.0*	28.7 ± 6.8*
	100x dilution	0.5 ± 15.2	6.4 ± 5.7
	1000x dilution	−2.7 ± 10.5	1.7 ± 5.6
	10,000x dilution	8.0 ± 11.0	2.4 ± 5.4
HM6- *Vuka Uphile*™ Immune Booster	Neat	42.6 ± 10.9*	39.6 ± 3.8*
	10x dilution	22.1 ± 9.6*	23.4 ± 6.0*
	100x dilution	1.5 ± 25.4	3.1 ± 9.5
	1000x dilution	7.3 ± 14.6	5.4 ± 7.9
	10,000x dilution	−9.5 ± 10.4	2.0 ± 5.8

Results presented as: mean ± standard deviation; * *p* ≤ 0.05, *n* = 8

## Discussion

In this study, the in vitro luminescence activity of platelets over 60 min was determined after being exposed to various concentrations of HMs. This is a direct measure of the amount of light emitted because of the production of ROS by the NADPH oxidase system when platelets are stimulated. PMA and fMLP were used to stimulate the platelets to elucidate the mechanisms by which the ROS production is affected. While the two agonists result in the activation of the NOX system by protein kinase C (PKC), PMA directly activates PKC whereas fMLP acts indirectly by binding to specific G-protein-linked formyl peptide receptors on the membrane. The light emitted was compared to that of the controls and was expressed as a percentage (%) inhibition. The higher the value, therefore, the more the inhibition on platelets, and the lower the value, the less the inhibition, with negative values indicating stimulation.

The results in this study showed a general weak inhibitory effect by the HMs on both fMLP- and PMA-induced platelets at both high and low concentrations. There was also a weak stimulatory effect at low concentrations for some of the HMs (Table 1). In the case of inhibition in the presence of the HMs, the NOX system that resulted in the production of ROS could not be activated, either directly through PKC activation or indirectly by receptor activation. Compounds in the HMs interacted with the PKC and with the formyl peptide receptors on the membrane resulting in less production of ROS. In the case of stimulation, which occurred at lower concentrations, the presence of HMs slightly enhanced (weak stimulation) the activity of the platelets via both pathways. It meant that the HMs interacted with PKC and the formyl peptide receptors such that the activity of the platelets was increased, resulting in more production of ROS as compared to the controls. The inhibition and stimulation effects hence translate to antioxidant and pro-oxidant activities of the HMs, respectively. This study, therefore, reports the in vitro inhibition and stimulation of platelets’ activity by CHMs used as ATM, mediated via the PKC and formyl peptide receptor pathways.

Many African medicinal plants have been shown to have antioxidant properties [15]. Several compounds isolated from the plant parts were confirmed for various activities, including the anti-inflammatory, antioxidant, and immunomodulatory activities [12, 16, 17]. Flavonoids are the plant compounds proven to have an inhibitory effect on the respiratory burst (ROS production) of cells [18].

Inhibition and stimulation of platelets imply anticoagulant and pro-coagulant effects by the HMs, respectively. Platelet ROS play a direct role in thrombosis [9], and platelet activation and platelet reactivity affect haemostasis and thrombosis [19]. Increased platelet ROS is implicated in the development, progression and complications of several disorders, including cardiovascular conditions such as hypertension, atherosclerosis, diabetes mellitus and stroke [9, 20]. By stimulating platelets, the HMs in this study, therefore, could inadvertently play a role in increasing the risk of thrombosis and the development of the cardiovascular and inflammatory conditions. Antiplatelet medicines are the cornerstone of the prevention and management of the mentioned conditions [21]. It has been suggested that plant extracts could serve as alternatives to antiplatelet drugs [22]. In inhibiting platelet activity and reducing ROS production, the tested HMs could, therefore, have a role to play in the prevention and attenuation of the diseases. These opposing effects signify the importance of appropriate dosing, which is key to differentiating the beneficial and unwanted effects. The inhibition of platelet activity by the HMS occurring at low doses could mimic the way antiplatelet drugs are used long-term at low doses for their effects. The risk of bleeding is a known complication of antiplatelet drugs [23] as well as some herbal medicines such as garlic, ginkgo, and ginger [22, 24]. Likewise, the weak inhibition of platelets by the HMs, which was largely concentration-dependent, could increase the risk of bleeding in individuals who take the HMs. It was stated that high and low levels of inhibition of platelets are closely linked to the risk of bleeding events and ischaemic events, respectively [23].

Regarding the immune system and inflammatory response, generally, activated platelets secrete chemical mediators that enhance the interaction of platelets with other cells of the immune system, including the recruitment of neutrophils to inflammatory tissue and the formation of aggregates with neutrophils, monocytes and lymphocytes [6, 8, 25, 26]. The platelet-neutrophil (P-N) complexes perform immune functions such as phagocytosis, cytotoxicity, and cytolysis more effectively than neutrophils on their own [27]. The presence of platelet-derived ROS increased the recruitment of neutrophils to a growing thrombus [9]. Platelet-derived ROS and other chemical substances directly destroy pathogens [9, 10]; hence, the weak stimulation effect on platelets could be one of the many mechanisms by which the HMs enhance the immune system. The inhibition of platelets by the HMs could therefore, contribute to the impairment of the immune system and inflammatory processes.

Platelet-neutrophil interactions were implicated in the development and progression of diseases such as myocardial ischaemia and atherosclerosis [28]. Furthermore, interactions among leucocytes, endothelial cells and platelets were reported to contribute to inflammation, and subsequently to the pathogenesis of various inflammatory diseases. Pathways of platelet ROS were found to be involved in the airway inflammation of allergic reactions. For these reasons, it was suggested that suppression of platelet activity could be an alternative mechanism for the management of allergic conditions [8].

Many herbal medicines have well-known antiplatelet effects, attributed to the different plant compounds, including polyphenols [21, 22]. Similarly, the antiplatelet activity of the HMs in this study would be attributed to the various phytochemicals that are present, although not specifically identified nor quantified. All the HMs in the study except HM4 are water-based concoctions, which intones the presence of polar compounds. HM4 is the only alcohol-based mixture, signifying the presence of a wider polarity range of compounds.

The extraction of polyphenols from plant material requires the use of aqueous mixtures of organic solvents such as acetone, acetonitrile, methanol, and ethanol [29, 30]. Ethanol is deemed the best solvent to extract polyphenols, and it is safe for human consumption. Aqueous mixtures of the solvents generally provide a higher extraction yield than water and the pure solvents. Aqueous ethanol has shown a higher extraction yield than water [30]. It, therefore, signifies that HM4 contains more polyphenols compared to the other water-based CHMs, which would explain its effects that were dissimilar to the other HMs. This deviant behaviour was also observed in another study by Mothibe et al. [31].

Several phytochemicals have been isolated in the medicinal plants contained in some of the CHMs. The inhibitory and stimulatory effects of HM1, HM2, HM4, and HM5 could be ascribed to the aloe component of the HMs, since aloe has been proven, in vitro that it had antioxidant, anti-inflammatory and immunostimulatory activity [12, 32]. Compounds that have been isolated in aloe include flavonoids, alkaloids and bioactive compounds such as aloin, aloesin and aloemodin, dithranol and magnesium lactate [33]. L-canavanine, GABA and D-pinitol are compounds isolated from Sutherlandia, which forms a component of HM2 and HM4. Compounds present in *H. hemerocallidea* (African potato) could include secondary metabolites such as glycosides, polyphenols, saponins, steroids, tannins and its main compounds, hypoxoside and rooperol. The activity of *Harpagophytum procumbens* (devil’s claw) present in HM4 could be attributed to its glycosides [12]. Echinacea, alfalfa-lucerne (*Medicago sativa*), and dandelion (*Taraxacum officinale*) are well-known Western herbal medicines used mainly for immune-boosting effects. Many phytochemicals have been identified and isolated from these plants, including tannins, saponins, flavonoids, terpenoids, and phenolic compounds, which were proven for anti-inflammatory, antioxidant, immunostimulant and antithrombotic activities [17, 34]. The effects of the CHMs on the platelets, therefore, could be ascribed to the many compounds acting in synergy, additively, and possibly antagonistically. Both the antioxidant and the pro-oxidant activities of the CHMs reported in this study provide some rationale for using these medicines for their intended purpose. In a healthy physiological system, the production of ROS and the endogenous antioxidant defence mechanisms are in equilibrium. Disturbance of this balance results in oxidative stress and its associated harmful effects which include damage to biological macromolecules, leading to various pathologies [35, 36].

Polyphenols have been reported to target and block various platelet activation pathways, and therefore, had the potential to replace or complement antiplatelet therapy [36]. This study reports on additional pathways that are potential targets of polyphenols in platelets, namely the intracellular PKC pathway and the membrane-bound G-protein linked formyl peptide receptor pathway. The findings are in agreement with the report by Santhakumar et al. [36] that the antioxidant effect of polyphenols is achieved by the blockage of different receptors on the platelet surface that are responsible for platelet activation, and thereby, eliminating free radicals.

Several biochemical pathways in platelets are regulated by PKC and affect platelet physiology [19] while ROS are involved in various signal transduction mechanisms [20]. The platelet-enhancing and platelet-inhibiting effects, which occurred at high and low concentrations of the HMs, signify the potential for variable effects depending on the dose of the HM. These HMs are used for self-medication as immune boosters and the effects, therefore, highlight the possibility of herb-drug interactions (HDIs). The HDIs could occur if the HMs are used concomitantly with conventional medicines, particularly antiplatelet medicines such as NSAIDs.

The lifespan of platelets in circulation is on average 10–14 days. Because of their effects, it is generally recommended that herbal medicines be stopped before any surgical procedure, with periods ranging from one to 2 weeks before [24, 37–39]. The safety of the CHMs is not well-defined; however, based on the results obtained, it is recommended that using the CHMs in this study be stopped 14 days before any surgical procedure. It is also crucial that the consumers and healthcare providers be aware of the possible effects and the potential of interactions with other medicines.

## Conclusion

The findings in this study show the in vitro antiplatelet and platelet-enhancing activities of CHMs used as ATMs at high and low concentrations. These activities were mediated via the intracellular PKC pathway and via the G-protein linked formyl peptide receptors on the membrane. Although the CHMs are commonly used as immune boosters, their diversity in composition renders their effects on platelets unpredictable. The findings suggest potential beneficial roles of the CHMs in the suppression of platelets’ reactivity and in enhancing the immune system. Caution, however, should be exercised, as platelet inhibition and stimulation predispose to the risk of bleeding and thrombosis, respectively.

## Data Availability

All the data that supported this article are presented in Figs. 2, 3, 4, 5, 6, 7 and Tables 1 and 2. The raw data sets that were analysed in the study are uploaded in the repository – Mendeley Data and is available at 10.17632/tvnyyz6t8w.1. The DOI is doi:10.17632/tvnyyz6t8w.1

## Abbreviations

% inh: Percentage inhibition
ATM/s: African traditional medicine/s
CHMs: Commercial herbal medicines/mixtures
CL: Chemiluminescence
EDTA: Ethylene-diamine-tetra-acetic acid
fMLP: N-formyl-methionyl-leucyl-phenylalanine
H_2_O_2_: Hydrogen peroxide
HBSS: Hanks’ balanced salt solution
HDIs: Herb-drug interactions
HM/s: Herbal medicine/s
LEL: Luminol-enhanced luminescence
NADPH: Nicotinamide adenine dinucleotide phosphate
NOX: NADPH oxidase
NSAIDs: Non-steroidal anti-inflammatory drugs
O_2_^−^: Superoxide anion
OH^−^: Hydroxyl radical
PKC: Protein kinase C
PMA: Phorbol myristate acetate
PRP: Platelet-rich plasma
RLU: Relative luminescence units
ROS: Reactive oxygen species
rpm: Revolutions per minute
SA: South Africa
SMUREC: Sefako Makgatho Health Sciences University Research and Ethics Committee
TM/s: Traditional medicine/s
